# Editorial: Plasticity of monocytes/macrophages: phenotypic changes during disease progression

**DOI:** 10.3389/fimmu.2023.1328382

**Published:** 2023-11-13

**Authors:** Ruoxi Yuan, Yanan Ma, Chao Yang, Liwu Li

**Affiliations:** ^1^ HSS Research Institute and David Z. Rosensweig Genomics Research Center, Hospital for Special Surgery, New York, NY, United States; ^2^ Memorial Sloan Kettering Cancer Center, New York, NY, United States; ^3^ Center for Immunological and Metabolic Diseases, Med-X Institute, First Affiliated Hospital of Xi’an Jiaotong University, Xi’an Jiaotong University, Xianyang, Shaanxi, China; ^4^ Department of Biological Sciences, Virginia Tech, Blacksburg, VA, United States

**Keywords:** macrophage heterogeneity, monocytes polarization, innate immunity, inflammation, therapeutic targets

Monocytes and macrophages, with their inherent heterogeneity, manifest a spectrum of phenotypes and functions finely tuned by their surrounding microenvironments. These versatile immune sentinels exhibit a remarkable capacity to adapt their responses within distinct pathological contexts, thus positioning them as central characters in various medical conditions. In summarizing the recent advances in our understanding of monocyte and macrophage heterogeneity in regulating disease progression and maintaining tissue homeostasis, we have compiled research articles encompassing various aspects of macrophage polarization and its impact within this specific Research Topic.

A more comprehensive understanding of the molecular mechanisms governing the generation, activation, and polarization of macrophages is imperative as a fundamental requirement for devising innovative therapeutic approaches to modulate macrophage functions within pathological contexts. Our Research Topic includes three reviews, each approaching this subject from distinct angles, focusing on autoimmune diseases (Yang et al.), adult hippocampal neurogenesis (Fang et al.), and human malignancies (Chaintreuil et al.). Additionally, it is noteworthy that advancements in cutting-edge technologies, such as single-cell RNA sequencing, which has also been utilized by papers under this topic as well, have paved the way for significant breakthroughs in the field of monocytes and macrophages.

Cong et al. summarized the advanced awareness of the multifaceted roles played by macrophages in the regulation of aseptic loosening (AL) pathogenesis. AL, the most common complication of total joint arthroplasty, is associated with activated macrophages that produce proinflammatory mediators, subsequently triggering the activation of osteoclasts, leading to bone breakdown. Additionally, macrophages, present in both homeostatic and injured skeletal muscle tissues, encompass heterogeneous functional subtypes that perform diverse roles in maintaining homeostasis and facilitating injury repair. Li et al. identified five distinct monocyte/macrophage subpopulations during intervertebral disk degeneration (IDD) using a single-cell RNAseq dataset spanning early to late degenerative stages of the intervertebral disk (IVD). The authors suggested that selectively removing regulatory macrophages in the early stage and oxidative stress (OS)-related macrophages in the late stage could alleviate angiogenesis and promote IDD recovery. Furthermore, genetic engineering of macrophages has been highly appreciated in numerous therapeutic approaches. Liang et al. utilized engineered L-M2a macrophages displaying a typical anti-inflammatory phenotype akin to M2 macrophages *in vitro*, resulting in markedly enhanced therapeutic outcomes for osteoarthritis (OA) by effectively addressing inflammation, reinstating tissue homeostasis, and promoting cartilage regeneration. Garabuczi et al. unveiled the pivotal roles of PPARg and Nur77 in shaping distinct macrophage subsets during skeletal muscle injury in a cardiotoxin–induced injury model.

The role of non-coding RNAs (ncRNAs), including long non-coding RNAs (lncRNAs) and microRNAs (miRNAs), in the regulation of macrophage polarization is an emerging field of interest, as comprehensively reviewed by Qiao et al. and Yu et al. The potential of lncRNAs as both biomarkers and therapeutic targets for modulating macrophage polarization during disease development is increasingly recognized. For example, Erdem et al. demonstrated the pivotal role of these small RNA molecules in finely tuning macrophage responses, particularly in inducing epigenetic modifications and miRNA levels changes upon exposure to cellulose nanocrystals (CNF) and multiwalled carbon nanotubes (MWCNT). Inhalation of nanomaterials has been associated with the induction of inflammation in the lungs. These nanomaterials prompt phenotypic alterations in alveolar macrophages, with CNF exposure enhancing the M1 phenotype and MWCNT promoting the M2 phenotype. The manipulation of ncRNA expression emerges as a novel approach to regulate macrophage polarization, thus influencing inflammation, fibrosis, immune responses, and even tumorigenesis.

Macrophages play a pivotal role in the delicate balance of immune regulation in the context of Inflammatory Bowel Disease (IBD), and their dysregulation can lead to inflammation and tissue damage. Wang et al. summarized updated therapeutic approaches targeting macrophage polarization in Ulcerative Colitis, and concurrently, Chauvin et al. conducted original research demonstrating that NOD2 negatively regulates a macrophage developmental program through a feed-forward loop. This finding offers promise for addressing resistance to anti-TNF therapy in Crohn’s Disease.

Although monocytes and macrophages that migrate to sites of injury or inflammation have received significant attention in research, it’s equally important to acknowledge that tissue-resident macrophages like Kupffer cells, microglia, and Hofbauer cells are subjected to environment-mediated polarization as well. These resident macrophages are intricately involved in local inflammation and reparative processes within their respective tissues. Mercnik et al. showed that the inflammatory environment of preeclampsia (PE) causes the phenotypic changes observed between early and late PE Hofbauer cells (HBCs). Furthermore, the role of Kupffer cells in Non-alcoholic fatty liver disease (NAFLD) was also well documented in reviews by Xiao et al. and Yu et al. Microglia, the sole macrophage population within the central nervous system, holds the remarkable capability to modulate adult hippocampal neurogenesis in the context of depression. The findings summarized by Fang et al. underscore the potential for pharmaceutical interventions to specifically target microglia as a promising strategy for the treatment of depression.

As highlighted in this discussion, this Research Topic encompasses a wide array of seminal articles delving into the intricate mechanisms governing the shift of macrophage phenotypes at various stages of disease progression. It offers a comprehensive overview of potential therapeutic targets and approaches for modulating macrophage behavior in the treatment of these conditions.

## Author contributions

RY: Writing – original draft. YM: Writing – review & editing. CY: Writing – review & editing. LL: Writing – review & editing.

